# Transcriptomic Profiling Reveals Early Molecular Adaptations to Resistance Exercise During Disuse Muscle Atrophy with Complementary Automated H&E-Based Muscle Morphometry

**DOI:** 10.3390/life16091403

**Published:** 2026-08-25

**Authors:** Hub E Fatima, Chien-Lin Lin, Yu-Jung Cheng

**Affiliations:** 1 PhD Program in Healthcare Science, College of Healthcare Science, China Medical University, Taichung 406040, Taiwan; 2 Department of Physical Medicine and Rehabilitation, Asia University Hospital, Asia University, Taichung 413505, Taiwan; 3 Department of Physical Medicine and Rehabilitation, China Medical University Hospital, Taichung 404328, Taiwan; 4 Department of Physical Therapy and Graduate Institute of Rehabilitation Science, China Medical University, Taichung 406040, Taiwan

**Keywords:** disuse muscle atrophy, resistance exercise, transcriptomics, muscle fiber morphology, quantitative morphometry

## Abstract

Although resistance exercise effectively attenuates disuse muscle atrophy, studies examining both quantitative muscle morphometry and transcriptomic responses during early adaptation remain limited. To comprehensively evaluate these adaptations, we performed automated Hematoxylin and Eosin stain (H&E)-based quantitative morphometry alongside transcriptomic profiling in a mouse model of hindlimb immobilization with or without resistance exercise. Resistance exercise partially restored muscle fiber atrophy and restored muscle morphology. Transcriptomic profiling identified 76 differentially expressed genes and revealed coordinated alterations involving metabolic regulation, immune/inflammatory responses, and extracellular matrix remodeling, rather than a single dominant signaling pathway. Functional enrichment analysis further highlighted cytokine-mediated signaling, extracellular matrix organization, and metabolic pathways associated with the early response to resistance exercise. Together, these findings demonstrate that automated H&E-based morphometry, alongside transcriptomic profiling, provides complementary structural and molecular insights into the early adaptive response to resistance exercise and offers a practical framework for future studies of skeletal muscle remodeling.

## 1. Introduction

Decreased demand is the cause of disuse muscle atrophy, such as prolonged immobilization [1], decreased ambulation [2] or exposure to hypogravity [3]. The reduced muscle mass occurs due to an imbalance between protein synthesis and degradation. Therefore, protein translation is an important regulator of muscle protein synthesis, especially during rapid muscle loss, when the protein synthesis rate decreases [4]. Previous studies have shown that inactivity markedly reduces protein synthesis in skeletal muscle, leading to rapid loss of muscle mass and function [5,6].

Resistance exercise is widely recognized as one of the most effective interventions for attenuating disuse muscle atrophy by restoring muscle mass and promoting functional recovery [7]. Previous studies have demonstrated its beneficial effects through either histological assessment of muscle fiber morphology [8] or transcriptomic analysis of gene expression [9]. However, these approaches have largely been applied independently, providing different perspectives on the structural and molecular responses occurring during the early phase of muscle recovery. Assessing both morphological and transcriptomic responses may therefore provide a broader characterization of the biological adaptations to resistance exercise.

Hematoxylin and eosin (H&E) staining is a widely used method for assessing skeletal muscle morphology [10]. Routine H&E staining restored overall tissue architecture and provides excellent visualization of myofiber morphology; however, objective quantitative analysis of H&E-stained sections remains technically challenging because individual fiber boundaries are often difficult to delineate automatically [11]. Consequently, quantitative muscle fiber analysis has traditionally relied on cryosections combined with laminin or dystrophin immunostaining to facilitate automated segmentation. Although these approaches provide accurate measurements of muscle fiber cross-sectional area, they require additional tissue processing and may compromise overall tissue morphology. Although recent studies have demonstrated the feasibility of AI-assisted analysis of H&E-stained skeletal muscle sections [12], studies incorporating both objective morphometric assessment and transcriptomic profiling to characterize early skeletal muscle adaptation remain limited. Conventional manual quantification of muscle fiber morphology is labor-intensive and subject to inter- and intra-observer variability. Recent advances in automated image analysis, such as deep learning-based segmentation, enable rapid, objective, and reproducible assessment of muscle fiber cross-sectional area, improving the reliability and efficiency of histological analysis. In the present study, we performed automated H&E-based quantitative morphometry alongside transcriptomic profiling to characterize the structural and molecular responses to resistance exercise following disuse muscle atrophy.

## 2. Materials and Methods

### 2.1. Study Design

**Figure 1:**
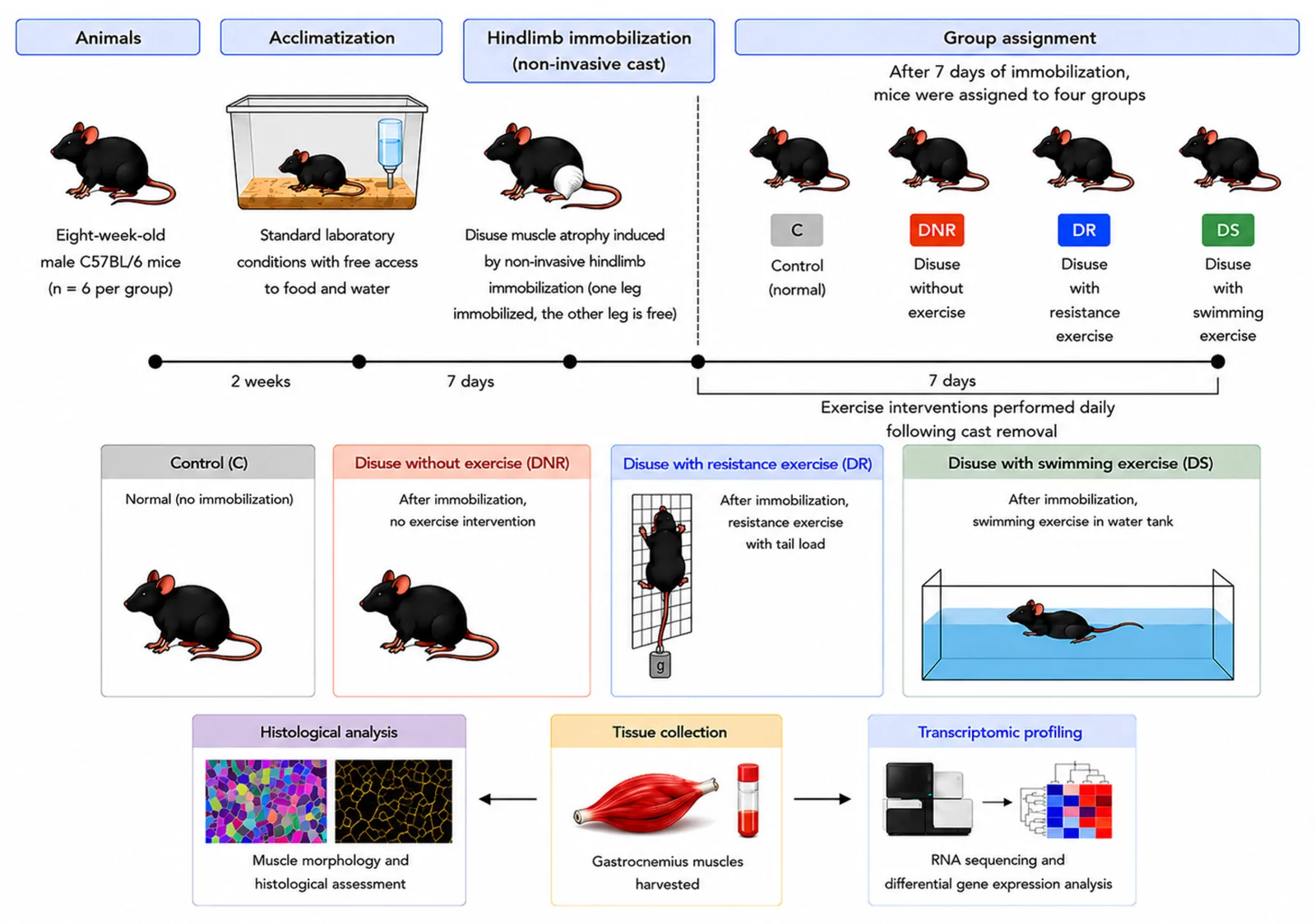
Schematic illustration of the experimental design.

Eight-week-old male C57BL/6 mice were housed under standard laboratory conditions with free access to food and water. Following a two-week acclimatization period, disuse muscle atrophy was induced using a previously described non-invasive hindlimb immobilization model [13]. After seven days of immobilization, mice (6 per group) were assigned to the control (C), disuse without exercise (DNR), disuse with resistance exercise (DR), or disuse with swimming exercise (DS) groups. Exercise interventions were performed daily for seven days following cast removal. Gastrocnemius and tibialis anterior muscles were harvested for histological analysis and transcriptomic profiling. The overall experimental design is illustrated in Figure 1.

### 2.2. Exercise Intervention

Resistance exercise was performed as adapted from previous study [14]. For resistance exercise intervention, each mouse was weighed. A 50 mL centrifuge tube containing iron balls was prepared so that the total weight equaled 85% of body weight after immobilization was removed. Under light 3% isoflurane anesthesia, the tube was secured to the tail with paper tape. The mouse was then placed on a metal mesh grid (1 × 1 cm), which was held vertically, once the mouse was awake and able to grip, allowing it to climb. After reaching the top, the grid was inverted to continue climbing. This exercise was performed for 5 min. The load on mouse tail was increased to 100% on day 2, and training was continued daily for 7 days.

In swimming training, each mouse was weighed and placed in a water tank (15 cm depth, 30 °C) for swimming exercise after cast removed. On day 1, the mice swam for 15 min to adapt to the protocol, and the duration was increased to 30 min from day 2 onward. Training was performed daily for 7 days. After each session, the mice were dried under an infrared heat lamp.

### 2.3. Tissue Collection and Histological Analysis

After 7 days of exercise training, six mice per group (n = 6) were anesthetized with 3% isoflurane, and blood was collected by cardiac puncture. The mice were then sacrificed by cervical dislocation. The hind limbs were sprayed with 70% alcohol, and the skin was cut at the ankle and reflected upward to expose the Achilles tendon and lower limb muscles. After removing the surrounding fascia, the Achilles tendon was cut, and the gastrocnemius, soleus muscles and tibialis anterior muscle were carefully dissected and weighed using a microscale. To maintain tissue integrity for histological assessment, the gastrocnemius muscle was dedicated to morphological analysis, while the tibialis anterior muscle was used for RNA sequencing, as transcriptomic analysis requires complete tissue homogenization.

Histological analysis was performed using four biological samples per group, according to the predefined tissue-embedding and slide-preparation protocol, in which four muscle specimens were processed per block/slide. After the muscle tissue was fixed in 4% formalin solution, it was dehydrated and embedded in paraffin. After the tissue wax blocks were sectioned into 4 μm thick sections, the slides were placed in an iron rack in the same orientation and baked in a 70 °C oven for 10–20 min. The sections were then deparaffinized in xylene and rehydrated through a graded ethanol series from 99.5% to 70%. The rehydrated tissue was stained with hematoxylin for 30 s and rinsed under tap water for 20 min. The tissue sections were soaked in Eosin-Y for 8 min, then dehydrated with 70% to 99.5% alcohol, and finally sealed with sealant.

### 2.4. Automated H&E-Based Quantitative Morphometry

H&E-stained gastrocnemius muscle sections were digitized using a light microscope equipped with a digital imaging system. Histological analysis was performed using sections from four mice per group (n = 4). To minimize anatomical variation, one cross-sectional image was acquired from the standardized central region of the gastrocnemius muscle in each specimen. All images underwent identical preprocessing procedures before automated segmentation. A customized CellPose model was initialized from the pretrained cytomodel and trained using four representative H&E-stained images from the control and disuse groups, thereby including both normal-sized and atrophic muscle fibers. Initial segmentation masks were generated using the pretrained model and visually reviewed against the corresponding H&E images. Incorrectly segmented masks were manually removed, and the remaining curated masks were used as training annotations to develop the customized model. The resulting model was subsequently reapplied to the training images to assess segmentation performance before analysis of the study images.

**Figure 2:**
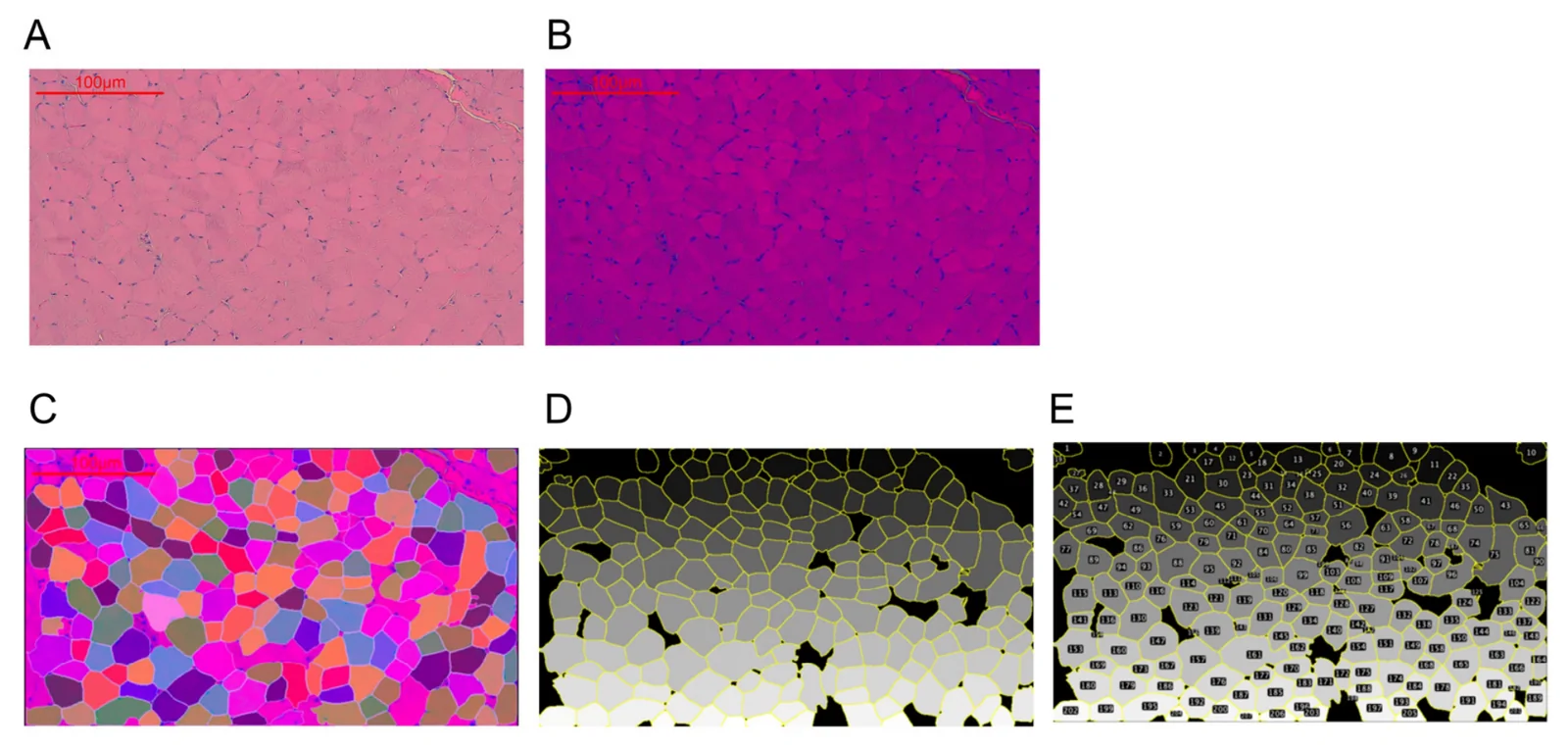
Workflow for skeletal muscle fiber cross-sectional area analysis using CellPose and Fiji/ImageJ. (**A**) Representative H&E-stained skeletal muscle section acquired using light microscopy. (**B**) Preprocessed image with enhanced contrast to improve visualization of muscle fiber boundaries. (**C**) Automated muscle fiber segmentation using the customized CellPose model. (**D**) Conversion of segmentation masks into regions of interest (ROIs) in Fiji/ImageJ. (**E**) ROI labeling and measurement of individual muscle fiber cross-sectional area.

For quantitative analysis, image-specific adjustment of segmentation parameters was performed when necessary to accommodate differences in muscle fiber size and staining characteristics. Segmentation was generally initiated using an estimated object diameter of 40 pixels; for images containing markedly smaller atrophic fibers, the diameter was reduced to 30 pixels when visual inspection indicated inadequate fiber delineation. The flow threshold was likewise adjusted according to segmentation performance. The resulting masks were imported into Fiji/ImageJ (1.51m9) and converted into regions of interest (ROIs) using the MorphoLibJ plugin. All ROIs were visually inspected against the corresponding H&E images. Incorrectly delineated ROIs were manually edited, whereas ROIs that could not be reliably corrected were excluded before quantitative analysis. Muscle fiber cross-sectional area was then measured from the finalized ROIs. The complete image-processing workflow is illustrated in Figure 2.

### 2.5. RNA Sequencing

Total RNA was extracted using TRIzol^®^ Reagent (Invitrogen, Carlsbad, CA, USA) following the manufacturer’s instructions. Chloroform was used for phase separation, with RNA recovered from the aqueous phase. The purified RNA was quantified at OD260 nm using an ND-1000 spectrophotometer (NanoDrop Technologies, Wilmington, NC, USA), and RNA quality was assessed using a Bioanalyzer 2100 with the RNA 6000 LabChip kit (Agilent Technologies, Santa Clara, CA, USA). RNA samples were obtained from tibialis anterior (TA) muscles from the four experimental groups (NR, R, DNR, and DR). Libraries were prepared using the SureSelect Strand-Specific RNA Library Preparation Kit (Agilent Technologies, Santa Clara, CA, USA), followed by size selection using AMPure XP beads (Beckman Coulter, Brea, CA, USA). RNA sequencing was performed by Welgene Biotech (Taipei, Taiwan) using Illumina sequencing-by-synthesis technology. FASTQ files were generated using a proprietary pipeline based on Illumina bcl2fastq v2.20. Adapter clipping and sequence quality trimming were performed using Trimmomatic (v0.36), and reads were aligned using HISAT2. Transcript assembly and quantification were performed using StringTie (v2.1.3).

Differential expression analysis between the disuse plus resistance exercise (DR) and disuse without resistance exercise (DNR) groups was performed using DESeq2 (v1.39.0). Multiple testing correction was performed using the Benjamini–Hochberg procedure to control the false discovery rate (FDR). Genes with an FDR-adjusted *q*-value < 0.05 and an absolute log2 fold change (|log2FC|) > 1 were considered significantly differentially expressed.

### 2.6. Transcriptomic Visualization and Functional Annotation

Principal component analysis (PCA) was performed using TPM expression values from all individual samples to evaluate global transcriptomic variation among the four experimental groups. A volcano plot was generated using all detected genes from the comparison between the DR and DNR groups. Genes with a false discovery rate (FDR)-adjusted *q*-value < 0.05 and an absolute log2 fold change (|log2FC|) > 1 were highlighted as significantly upregulated or downregulated.

Heatmaps were generated using TPM values from individual biological replicates of the four experimental groups (NR, R, DNR, and DR). Gene expression values were log2-transformed [log2(TPM + 1)] and standardized using row-wise Z-score normalization. Candidate genes were initially selected from significantly differentially expressed genes identified between the DR and DNR groups (FDR-adjusted *q*-value < 0.05 and |log2 fold change| > 1). Predicted and uncharacterized genes, as well as genes with low expression abundance or high inter-sample variability within the DR and DNR groups, were excluded. Hierarchical clustering was performed using Euclidean distance and complete linkage to identify genes with similar expression patterns across the four experimental groups. Functional annotations were displayed as row annotations for descriptive purposes and were not used to determine the clustering order.

Gene Ontology (GO) enrichment analysis, including biological process (BP), molecular function (MF), and cellular component (CC), together with Kyoto Encyclopedia of Genes and Genomes (KEGG) pathway enrichment analysis, was performed using ClusterProfiler (v3.6). Enrichment significance was evaluated using the hypergeometric test, and multiple testing correction was performed using the Benjamini–Hochberg (BH) procedure. The complete list of differentially expressed genes meeting the predefined significance criteria (FDR-adjusted *q*-value < 0.05 and |log2FC| > 1) is provided in Supplementary Tables S2–S4.

### 2.7. Statistical Analysis

Data are presented as mean ± standard deviation. Statistical analyses were performed using GraphPad Prism 9. Differences between groups were assessed using the Mann–Whitney test. Differences among the experimental groups were analyzed using the Kruskal–Wallis test followed by Dunn’s multiple comparisons test. A *p* value < 0.05 was considered statistically significant.

## 3. Results

### 3.1. Resistance Exercise Attenuated Muscle Loss

**Figure 3:**
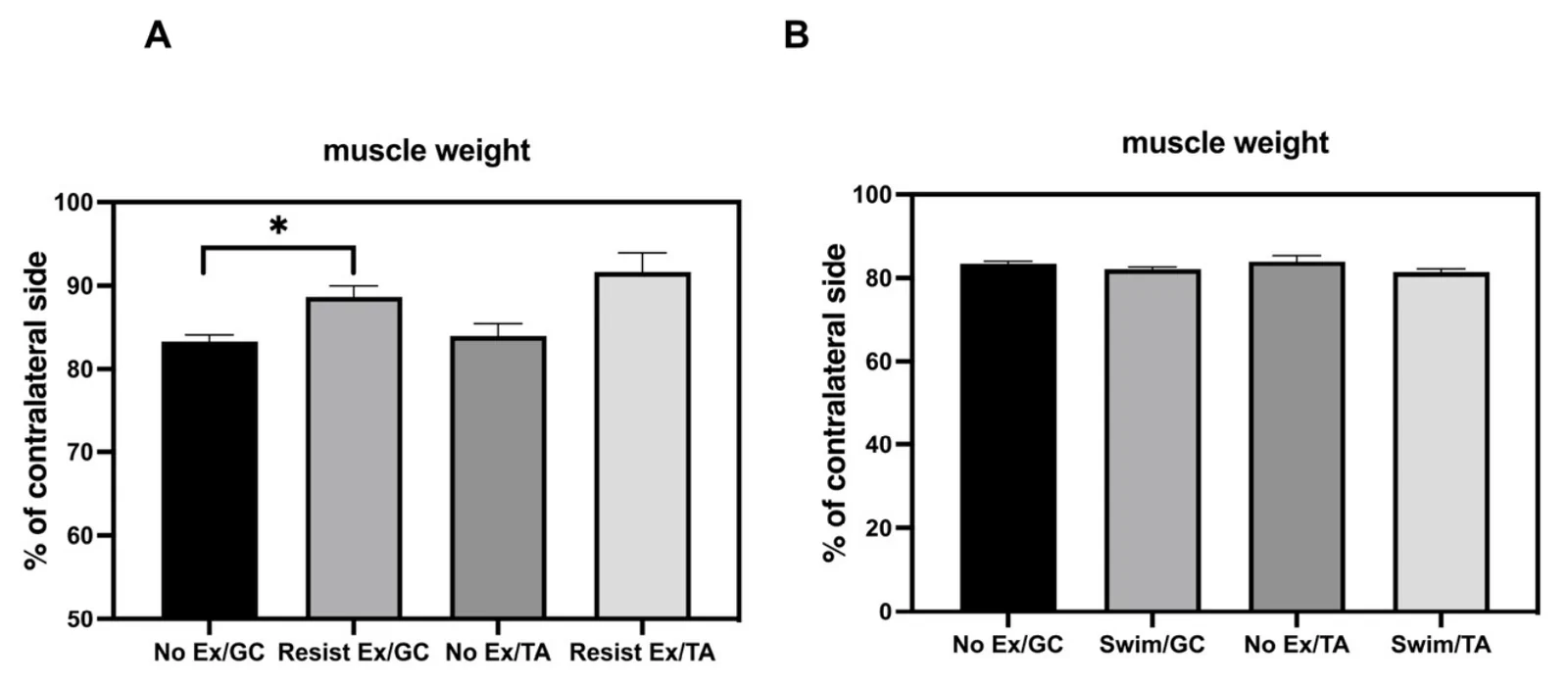
Changes in muscle weight following resistance exercise and swimming in the gastrocnemius and tibialis anterior compared to no exercise (**A**) Resistance exercise. (**B**) Swimming exercise. No Ex, no exercise; GC, gastrocnemius; Resist Ex, resistance exercise; TA, tibialis anterior; * *p* < 0.05.

Mice in the resistance training group (n = 6) performed 5 min of weighted climbing daily, whereas mice in the swimming group (n = 6) underwent 30 min of swimming each day. After 7 days of training, the animals were sacrificed and the gastrocnemius and tibialis anterior muscles were collected for weight measurement. Figure 3 shows resistance training was associated with changes in the gastrocnemius muscle that corresponded with differences in muscle weight following disuse atrophy, with a similar trend observed in the tibialis anterior. In contrast, swimming training did not produce an obvious beneficial effect, as the weights of both muscles remained similar to those in the non-exercise group. Terminal body weight at sacrifice did not differ significantly among the experimental groups (Supplementary Table S1).

### 3.2. Resistance Exercise Restored Muscle Fiber Morphology

**Figure 4:**
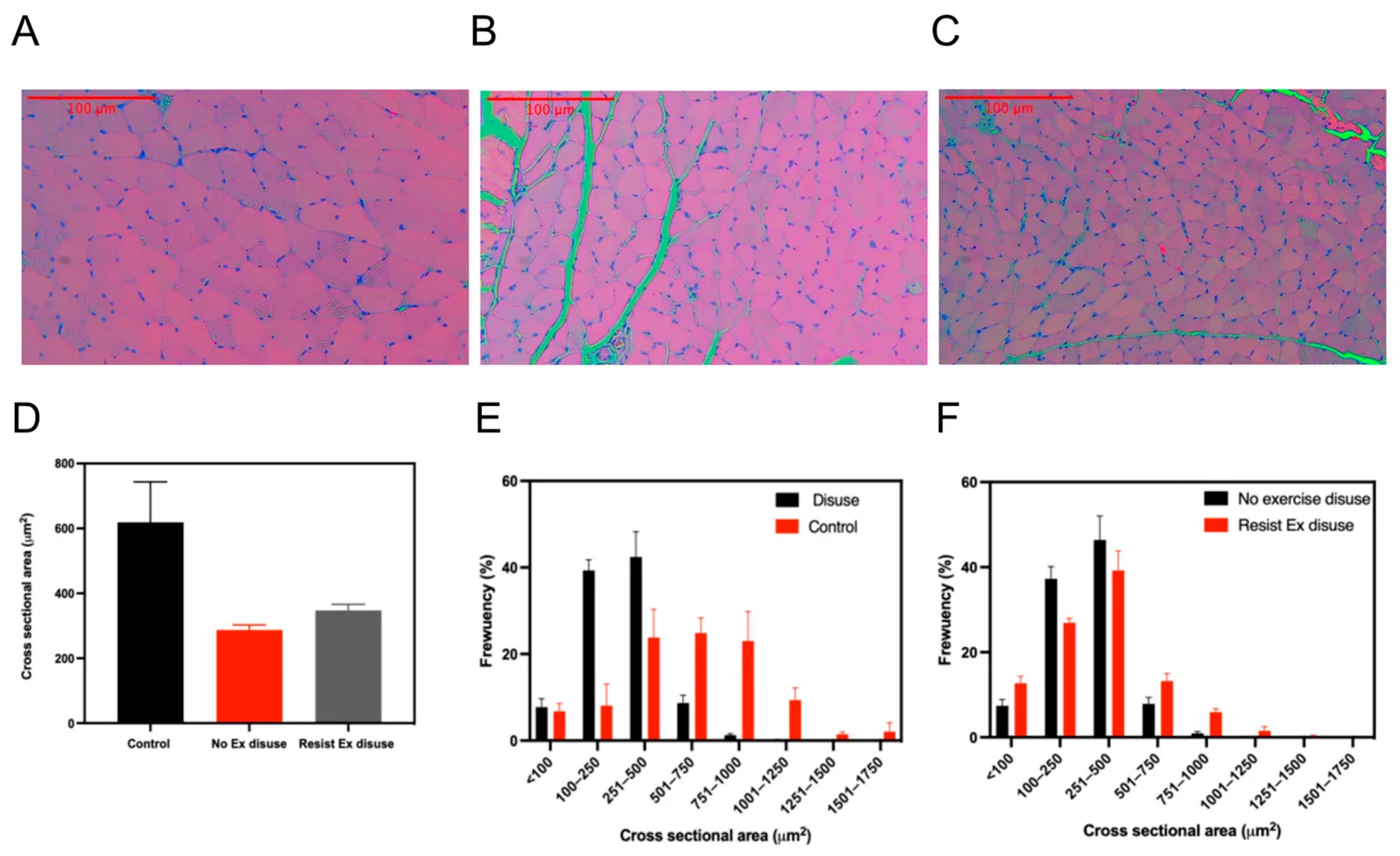
Post-exercise morphological adaptations following resistance exercise during recovery from disuse-induced muscle atrophy. (**A**–**C**) Representative H&E-stained gastrocnemius muscle sections from the control, disuse without exercise, and disuse with resistance exercise groups, respectively, collected after the 7-day intervention period. (**D**) Mean muscle fiber cross-sectional area (CSA) among the three groups. (**E**) Fiber-size distribution in the control and disuse without exercise groups, showing a shift toward smaller fibers following disuse. (**F**) Fiber-size distribution in the disuse without exercise and disuse with resistance exercise groups, showing a partial shift toward larger fibers following resistance exercise. Ex, exercise.

To further examine the effect of resistance training, the gastrocnemius muscles of resistance exercise group were collected after 7 days. The tissues were fixed in formalin, embedded in paraffin, sectioned, and stained with hematoxylin and eosin to assess changes in muscle fiber cross-sectional area. H&E-based quantitative morphometry demonstrated that 7 days of immobilization markedly reduced muscle fiber CSA, with a greater proportion of smaller fibers observed in the disuse atrophy group. Resistance exercise partially restored muscle fiber size, accompanied by a rightward shift in the CSA distribution, indicating restoration of larger muscle fibers following exercise (Figure 4). As a supplementary methodological assessment, CellPose-derived CSA measurements from H&E-stained sections were compared with those obtained from dystrophin-stained sections. The two staining approaches produced broadly comparable fiber-size distributions, and section-level mean CSA values were strongly correlated (r = 0.969, *p* = 0.016; Supplementary Figure S1).

### 3.3. Global Transcriptomic Profiling Identified Differential Gene Expression Following Resistance Exercise

**Figure 5:**
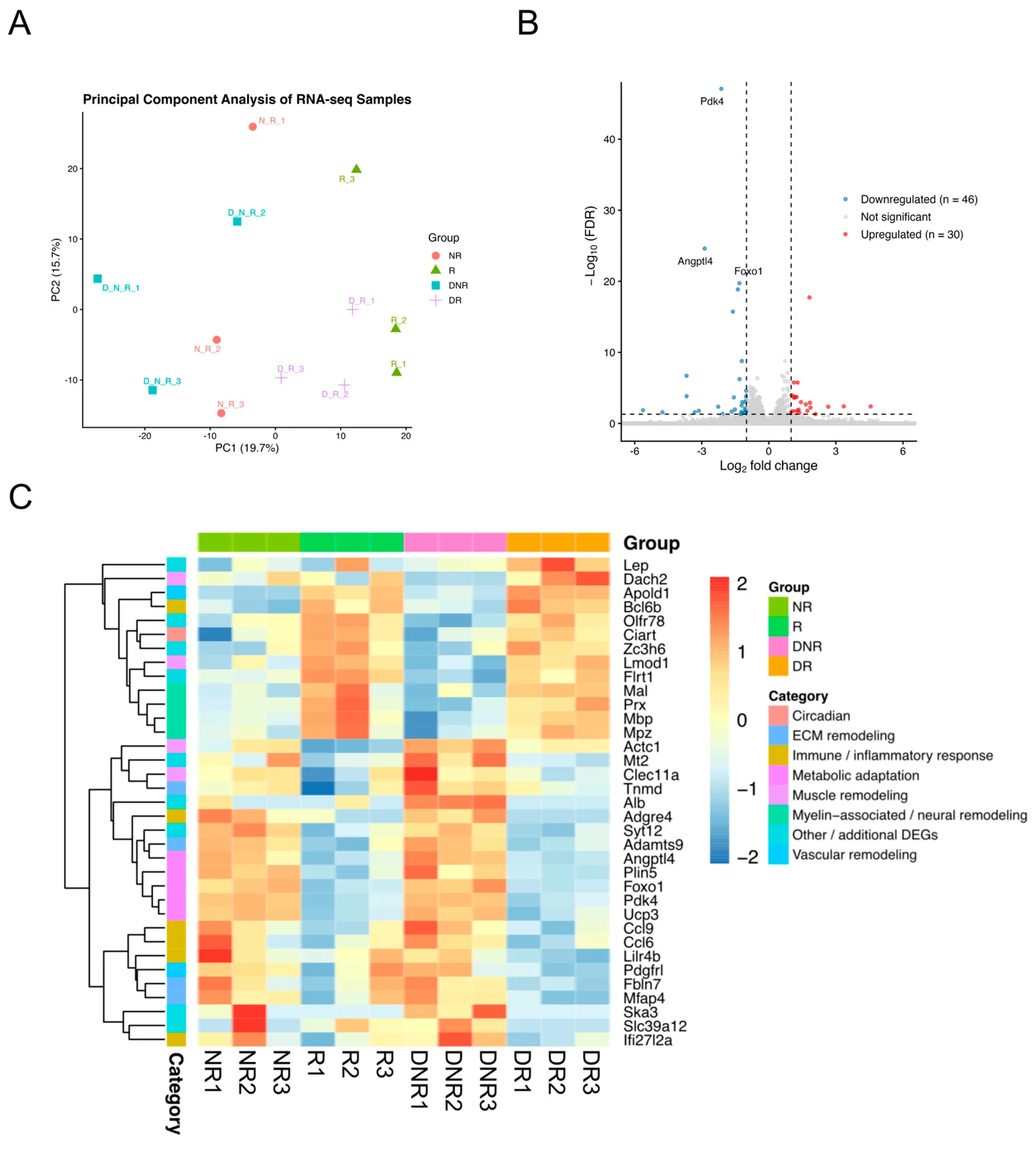
Transcriptomic profiling of skeletal muscle following resistance exercise during disuse. (**A**) Principal component analysis of RNA-seq samples from the four experimental groups. (**B**) Volcano plot of differentially expressed genes between the DNR and DR groups; red and blue indicate upregulated and downregulated genes, respectively. The vertical dashed lines indicate the fold-change threshold (|log2FC| = 1), and the horizontal dashed line indicates the significance threshold (FDR-adjusted *q*-value = 0.05). (**C**) Heatmap showing expression patterns of 35 selected genes across individual biological replicates from the NR, R, DNR, and DR groups (n = 3 per group). Expression values were log2-transformed [log2(TPM + 1)] and standardized by row-wise Z-score normalization. Functional categories are indicated by the row annotation.

Following immobilization to induce muscle atrophy, mice underwent one week of resistance training. The tibialis anterior muscle (3 mice per group) from the atrophic limb was then collected and submitted for next-generation RNA sequencing to assess gene expression differences after resistance training. Principal component analysis (PCA) demonstrated clear separation among the four experimental groups, indicating distinct global transcriptional profiles (Figure 5A). The DR samples were shifted away from the DNR group and showed partial overlap with the non-disuse groups, suggesting that resistance exercise partially reversed the transcriptomic alterations induced by disuse.

Differential expression analysis between the DR and DNR groups identified 76 significantly regulated genes (46 downregulated and 30 upregulated; FDR-adjusted q < 0.05 and |log2FC| > 1) (Figure 5B). Among the most significantly altered transcripts were the metabolic regulators Pdk4, Foxo1, and Angptl4, highlighting prominent changes in metabolic adaptation following resistance exercise. The complete list of differentially expressed genes is provided in Supplementary Tables S2–S4.

Hierarchical clustering of representative DEGs demonstrated distinct expression patterns across the four experimental groups. Visualization of individual biological replicates showed generally consistent expression trends within each experimental group despite expected biological variability (Figure 5C). Genes associated with metabolic adaptation (*Foxo1*, *Pdk4*, *Plin5*, *Ucp3*, and *Angptl4*) formed a coherent cluster and exhibited increased expression in the disuse group, whereas resistance exercise largely attenuated these transcriptional changes. Similar coordinated expression patterns were also observed for genes involved in immune/inflammatory responses (*Adgre4*, *Ccl6*, and *Ccl9*) and myelin-associated remodeling (*Mal*, *Mbp*, *Mpz*, and *Prx*), suggesting that resistance exercise modulated multiple biological programs beyond metabolic adaptation. To further assess the expression patterns of selected genes identified from the transcriptomic analysis, qPCR was performed using the R group as the reference. Among the 13 examined genes, nine showed directional changes consistent with the RNA-seq expression patterns, whereas four showed discrepant trends (Supplementary Figure S2).

### 3.4. Functional Enrichment Analysis Revealed Biological Pathways Associated with Resistance Exercise

**Figure 6:**
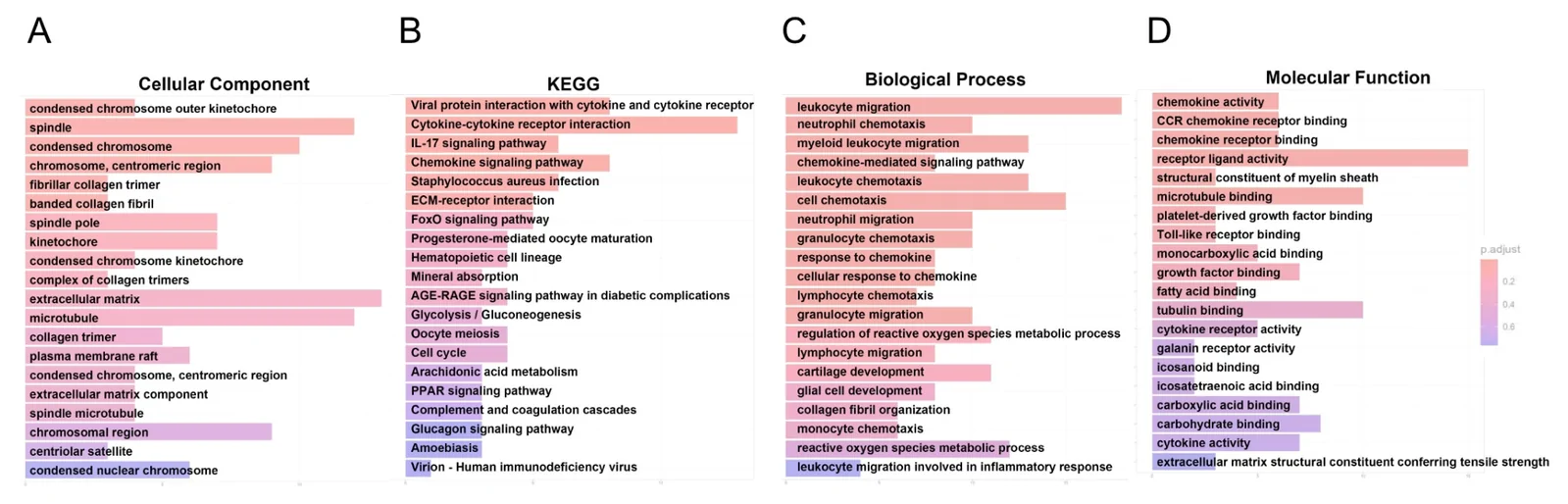
Functional enrichment analysis of differentially expressed genes: (**A**) cellular pathway, (**B**) kEGG pathway, (**C**) Biological pathway, and (**D**) Molecular pathway. Colors represent adjusted *p*-values (p.adjust), with warmer colors indicating greater statistical significance.

Functional enrichment analysis consistently indicated that resistance exercise modulated pathways involved in extracellular matrix remodeling, cytoskeletal organization, immune regulation, and metabolic adaptation. Specifically, CC and MF analyses highlighted extracellular matrix-, microtubule-, and cytokine-related functions, whereas KEGG analysis demonstrated enrichment of cytokine–cytokine receptor interaction, chemokine signaling, IL-17 signaling, ECM-receptor interaction, FoxO signaling, and metabolism-associated pathways, including glycolysis/gluconeogenesis and PPAR signaling. Consistent with these findings, BP analysis identified biological processes related to leukocyte migration, chemotaxis, reactive oxygen species metabolism, and inflammatory cell recruitment (Figure 6A–D).

## 4. Discussion

The present study demonstrated that short-term resistance exercise partially attenuated disuse-induced skeletal muscle atrophy and was accompanied by coordinated transcriptomic remodeling during early recovery. Using quantitative muscle morphometry together with transcriptomic profiling, we characterized structural and molecular responses to resistance exercise during disuse. The findings suggest that early recovery involves coordinated remodeling across multiple biological processes, rather than modulation of a single pathway.

Histological analysis showed partial restoration of gastrocnemius muscle fiber CSA and a shift in the fiber-size distribution toward larger fibers following resistance exercise. Although restoration of larger muscle fibers may indicate greater force-generating potential [15], muscle strength depends on multiple structural and neural factors and was not directly assessed in the present study. Therefore, these findings should be interpreted as evidence of structural restoration, rather than functional recovery.

Interestingly, resistance exercise increased muscle weight, whereas swimming did not produce a comparable effect. This difference may reflect the greater mechanical loading provided by resistance exercise and its stronger effect on restoration of muscle mass [16,17,18]. Because histological, molecular, and functional outcomes were not assessed in the swimming experiment, its potential protective effects against the disuse phenotype remain uncertain.

Among the transcriptomic alterations identified in this study, genes involved in metabolic adaptation exhibited one of the most consistent expression patterns across the four experimental groups. Hierarchical clustering revealed that metabolic regulators, including *Foxo1*, *Pdk4*, *Angptl4*, *Plin5*, and *Ucp3*, exhibited remarkably similar expression profiles across the four experimental groups. Notably, these genes exhibited lower expression levels in both exercise groups (R and DR) than in their corresponding non-exercise controls (NR and DNR), suggesting that resistance exercise elicited a conserved metabolic transcriptional response irrespective of disuse status. This observation suggests that resistance exercise modulates metabolic gene expression beyond the context of disuse recovery and may represent a fundamental adaptive response of skeletal muscle to mechanical loading.

These findings are biologically plausible, as FOXO1 is a well-established regulator of proteolysis and skeletal muscle atrophy, whereas PDK4 promotes a metabolic shift from glucose oxidation toward increased fatty acid utilization during catabolic conditions [19,20]. ANGPTL4 has likewise been implicated in lipid metabolism and exercise-induced metabolic adaptation in skeletal muscle [21]. Collectively, these findings suggest that resistance exercise consistently modulated the expression of key metabolic regulators under both non-disuse and disuse conditions. Collectively, these results support the concept that metabolic remodeling represents an early molecular response to resistance exercise during recovery from disuse-induced muscle atrophy.

In addition to metabolic remodeling, resistance exercise was associated with coordinated regulation of genes involved in immune responses and extracellular matrix remodeling. Hierarchical clustering revealed that representative inflammatory genes, including *Adgre4*, *Ccl6*, *Ccl9*, *Lilr4b*, and *Ifi27l2a*, exhibited remarkably similar expression profiles across the four experimental groups. A comparable pattern was observed for extracellular matrix-related genes, including *Adamts9*, *Fbln7*, *Mfap4*, and *Tnmd*, suggesting coordinated regulation of tissue remodeling pathways following resistance exercise. These observations were further supported by GO and KEGG enrichment analyses, which identified significant enrichment of cytokine-mediated signaling, leukocyte migration, chemotaxis, extracellular matrix organization, and ECM–receptor interaction.

The histological analyses demonstrated that resistance exercise partially restored gastrocnemius muscle fiber cross-sectional area and shifted the fiber-size distribution toward that of the non-disuse groups, indicating structural recovery following immobilization. In parallel, transcriptomic analysis of the tibialis anterior identified coordinated regulation of genes involved in extracellular matrix remodeling, muscle remodeling, and metabolic adaptation. These findings provide complementary evidence that short-term resistance exercise is associated with both structural and molecular adaptations during early recovery from disuse. However, because these analyses were performed in different muscles, the transcriptomic changes should not be interpreted as direct molecular correlates of the morphological improvements observed in the gastrocnemius.

In addition to the biological findings, the present study demonstrates the feasibility of combining conventional H&E histology with deep learning-based automated morphometric analysis for objective evaluation of skeletal muscle remodeling. Although laminin or WGA immunostaining is widely used for automated myofiber segmentation [22], these approaches require additional staining procedures and increase experimental cost and complexity. By contrast, our custom-trained CellPose model enabled accurate segmentation of H&E-stained muscle sections, allowing reproducible quantification of muscle fiber cross-sectional area while retaining the histological information required for routine pathological assessment [23].

Rather than replacing conventional histological evaluation, this workflow complements routine H&E staining by providing objective, reproducible, and scalable morphometric analysis. Because H&E staining remains the standard histological method in many experimental laboratories, this approach may facilitate future studies requiring both pathological interpretation and quantitative assessment without additional immunofluorescent staining.

Several limitations of the present study should be acknowledged. First, transcriptomic profiling was performed using tibialis anterior muscles, whereas histological analyses were conducted on gastrocnemius muscles. The gastrocnemius and tibialis anterior are functionally antagonistic muscles, contributing predominantly to plantarflexion and dorsiflexion of the hindfoot, respectively. This functional distinction represents an additional limitation when comparing responses between the two muscles. Muscle-specific differences between the gastrocnemius and tibialis anterior therefore preclude direct structure–molecular associations between the two datasets. The gastrocnemius muscle was maintained intact for quantitative morphometric analysis to avoid disruption of the complete muscle cross-section required for reliable H&E-based fiber segmentation. Consequently, the transcriptomic findings should be interpreted as a complementary but distinct characterization of the molecular response to the experimental intervention, rather than as direct molecular correlates of the morphological changes observed in the gastrocnemius muscle. Muscle-specific differences between the gastrocnemius and tibialis anterior preclude direct structure–molecular associations between the two datasets. Second, RNA sequencing was performed on a limited number of biological replicates because of the high cost of next-generation sequencing. To improve the robustness of the transcriptomic findings, principal component analysis, hierarchical clustering, and quantitative PCR assessment of selected genes were performed. Nevertheless, the transcriptomic results should be interpreted as exploratory and require confirmation in larger molecular datasets. Third, the intervention was limited to one week and employed a relatively low exercise volume to accommodate the reduced functional capacity of the immobilized hindlimb during early recovery. Although this protocol provided a feasible mechanical loading stimulus, the findings may not be generalizable to longer-duration or progressively loaded resistance training programs. In addition, direct measures of motor function, such as grip strength or in vivo muscle-force assessment, were not included. Therefore, the present study cannot determine whether the observed changes in muscle weight, fiber morphology, and gene expression translated into functional recovery. Future studies incorporating time- and load-gradient designs and multidimensional outcomes integrating muscle morphology, molecular markers, and functional performance are warranted. Finally, although the custom-trained CellPose model enabled objective analysis of H&E-stained muscle sections, additional validation across different muscles, staining protocols, and experimental models will be necessary before broader application of this workflow.

## 5. Conclusions

In conclusion, short-term resistance exercise partially attenuated disuse-induced muscle atrophy and was associated with coordinated transcriptomic alterations involving metabolic, immune/inflammatory, and extracellular matrix-related processes. H&E-based automated morphometry, alongside transcriptomic profiling, provided complementary structural and molecular perspectives on the early recovery response. In addition, the CellPose-based workflow demonstrated the feasibility of objective quantitative morphometry using conventional H&E-stained sections. Longer-term studies incorporating progressive loading and functional outcomes are needed to determine whether these early adaptations translate into sustained muscle recovery.

## Data Availability

The original contributions presented in this study are included in the article. Further inquiries can be directed to the corresponding author.

## Supplementary Materials

The following supporting information can be downloaded at: https://www.mdpi.com/article/10.3390/life16091403/s1, **Supplementary Figure S1:** Comparison of H&E- and dystrophin-based muscle fiber morphometry. **Supplementary Figure S2:** qPCR validation of selected RNA-seq findings. **Supplementary Table S1:** The terminal body weights. **Supplementary Table S2:** The genes in the resistance exercise group (DR) had the greatest upregulation compared with muscles without exercise (DNR). **Supplementary Table S3:** The genes in the resistance exercise group (DR) had the greatest degree of downregulation compared with muscles without exercise (DNR). **Supplementary Table S4:** The influence of muscle- and inflammation-related genes in the resistance exercise group (DR) compared with the non-exercise group (DNR).

## Abbreviations

The following abbreviations are used in this manuscript:

CSA	Cross-Sectional Area
DEG	Differentially expressed genes
DR	Disuse with resistance exercise
DNR	Disuse without resistance exercise
NGS	Next-generation sequencing
NR	No disuse without resistance exercise control
R	No disuse with resistance exercise
ROI	Region of interest
PCA	Principal component analysis

## References

[1] McGlory C., Gorissen S.H.M., Kamal M., Bahniwal R., Hector A.J., Baker S.K., Chabowski A., Phillips S.M. (2019). Omega-3 fatty acid supplementation attenuates skeletal muscle disuse atrophy during two weeks of unilateral leg immobilization in healthy young women. FASEB J..

[2] Oikawa S.Y., Holloway T.M., Phillips S.M. (2019). The impact of step reduction on muscle health in aging: Protein and exercise as countermeasures. Front. Nutr..

[3] Riley D.A., Ellis S., Slocom G.R., Satyanarayana T., Bain J.L., Sedlak F.R. (1987). Hypogravity-induced atrophy of rat soleus and extensor digitorum longus muscles. Muscle Nerve Off. J. Am. Assoc. Electrodiagn. Med..

[4] Vary T.C., Kimball S.R. (1992). Sepsis-induced changes in protein synthesis: Differential effects on fast-and slow-twitch muscles. Am. J. Physiol.-Cell Physiol..

[5] Gibson J.N., Halliday D., Morrison W.L., Stoward P.J., Hornsby G.A., Watt P.W., Murdoch G., Rennie M.J. (1987). Decrease in human quadriceps muscle protein turnover consequent upon leg immobilization. Clin. Sci..

[6] Howard E.E., Pasiakos S.M., Fussell M.A., Rodriguez N.R. (2020). Skeletal muscle disuse atrophy and the rehabilitative role of protein in recovery from musculoskeletal injury. Adv. Nutr..

[7] Adams G., Haddad F., Bodell P., Tran P., Baldwin K. (2007). Combined isometric, concentric, and eccentric resistance exercise prevents unloading-induced muscle atrophy in rats. J. Appl. Physiol..

[8] Son J.S., Kim J.H., Kim H.J., Yoon D.H., Kim J.S., Song H.S., Song W. (2016). Effect of resistance ladder training on sparc expression in skeletal muscle of hindlimb immobilized rats. Muscle Nerve.

[9] Thompson J.M., West D.W.D., Doering T.M., Budiono B.P., Lessard S.J., Koch L.G., Britton S.L., Byrne N.M., Brown M.A., Ashton K.J. (2022). Effect of short-term hindlimb immobilization on skeletal muscle atrophy and the transcriptome in a low compared with high responder to endurance training model. PLoS ONE.

[10] Wang C., Yue F., Kuang S. (2017). Muscle histology characterization using H&E staining and muscle fiber type classification using immunofluorescence staining. Bio-Protocol.

[11] Reinbigler M., Cosette J., Guesmia Z., Jimenez S., Fetita C., Brunet E., Stockholm D. (2022). Artificial intelligence workflow quantifying muscle features on Hematoxylin–Eosin stained sections reveals dystrophic phenotype amelioration upon treatment. Sci. Rep..

[12] Yamaoka Y., Chan W.I., Seno S., Iwamori K., Fukada S.-I., Matsuda H. (2024). Quantifying the recovery process of skeletal muscle on hematoxylin and eosin stained images via learning from label proportion. Sci. Rep..

[13] Wu K.C., Lin H.W., Chu P.C., Li C.I., Kao H.H., Lin C.H., Cheng Y.J. (2023). A non-invasive mouse model that recapitulates disuse-induced muscle atrophy in immobilized patients. Sci. Rep..

[14] Horii N., Uchida M., Hasegawa N., Fujie S., Oyanagi E., Yano H., Hashimoto T., Iemitsu M. (2018). Resistance training prevents muscle fibrosis and atrophy via down-regulation of C1q-induced Wnt signaling in senescent mice. FASEB J..

[15] Maughan R.J., Watson J.S., Weir J. (1983). Strength and cross-sectional area of human skeletal muscle. J. Physiol..

[16] Adams G.R., Bamman M.M. (2012). Characterization and regulation of mechanical loading-induced compensatory muscle hypertrophy. Compr. Physiol..

[17] Swift J.M., Nilsson M.I., Hogan H.A., Sumner L.R., Bloomfield S.A. (2010). Simulated resistance training during hindlimb unloading abolishes disuse bone loss and maintains muscle strength. J. Bone Min. Res..

[18] Fluckey J.D., Dupont-Versteegden E.E., Montague D.C., Knox M., Tesch P., Peterson C.A., Gaddy-Kurten D. (2002). A rat resistance exercise regimen attenuates losses of musculoskeletal mass during hindlimb suspension. Acta Physiol. Scand..

[19] Sanchez A.M., Candau R.B., Bernardi H. (2014). FoxO transcription factors: Their roles in the maintenance of skeletal muscle homeostasis. Cell Mol. Life Sci..

[20] Furuyama T., Kitayama K., Yamashita H., Mori N. (2003). Forkhead transcription factor FOXO1 (FKHR)-dependent induction of PDK4 gene expression in skeletal muscle during energy deprivation. Biochem. J..

[21] Catoire M., Alex S., Paraskevopulos N., Mattijssen F., Evers-van Gogh I., Schaart G., Jeppesen J., Kneppers A., Mensink M., Voshol P.J. (2014). Fatty acid-inducible ANGPTL4 governs lipid metabolic response to exercise. Proc. Natl. Acad. Sci. USA.

[22] Waisman A., Norris A.M., Elias Costa M., Kopinke D. (2021). Automatic and unbiased segmentation and quantification of myofibers in skeletal muscle. Sci. Rep..

[23] Stringer C., Wang T., Michaelos M., Pachitariu M. (2021). Cellpose: A generalist algorithm for cellular segmentation. Nat. Methods.

