# Supplementary material for: Transcriptomic Profiling Reveals Early Molecular Adaptations to Resistance Exercise During Disuse Muscle Atrophy with Complementary Automated H&E-Based Muscle Morphometry
Source: Life (Basel). 2026 Aug 25;16(9):1403. doi: 10.3390/life16091403 (PMC13608562; doi:10.3390/life16091403)
Supplement: Supplementary file 1 [file life-16-01403-s001.zip › life-4453041-supplementary.pdf]

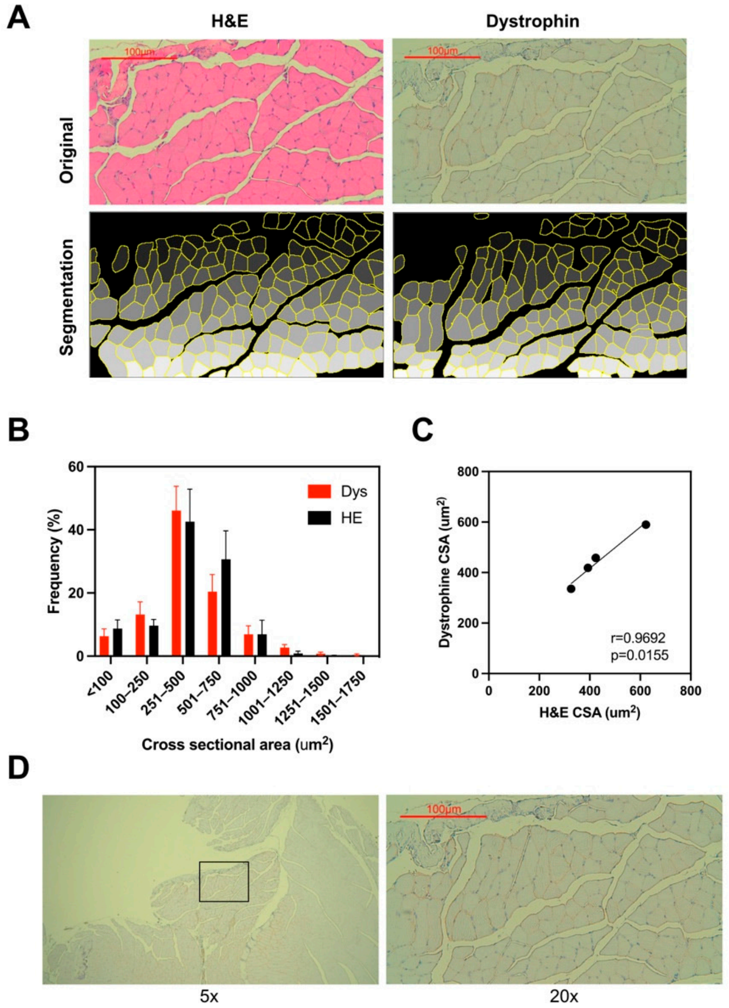

Supplementary Figure S1. Comparison of H&E- and dystrophin-based muscle fiber morphometry. (A) Representative original and CellPose-segmented images of H&E- and dystrophin-stained gastrocnemius muscle sections. (B) Comparison of muscle fiber cross-sectional area (CSA) distributions obtained from H&E- and dystrophin-stained sections. (C) Correlation between section-level mean CSA measured using H&E and dystrophin staining. (D) Representative dystrophin-stained section illustrating regional variation in staining quality at low (5×) and higher (20×) magnification.

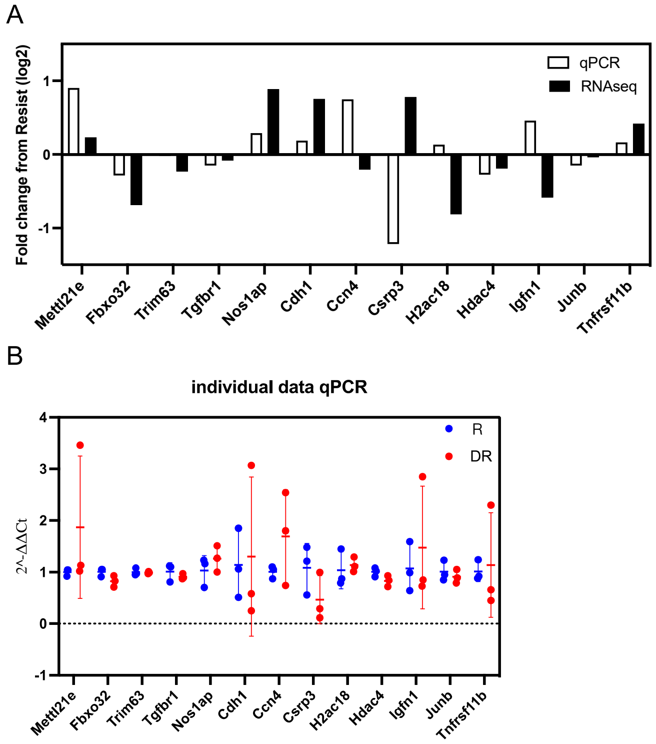

Supplementary Figure S2. qPCR validation of selected RNA-seq findings. (A) Comparison of log2 fold changes obtained by RNA-seq and qPCR for 12 selected genes. (B) Individual qPCR measurements in the R and DR groups. Each point represents an individual biological replicate (n = 3 per group). Target-gene Ct values were obtained from technical duplicates and normalized to GAPDH. Relative gene expression was calculated using the 2^−ΔΔCt method, with the R group used as the calibrator. Data are presented as mean ± SD.

Group	Terminal body weight (g)
No exercise	23.43 ± 0.92
Resistance exercise	23.90 ± 1.06
Swimming exercise	24.03 ± 1.09

Supplementary Table S1. The terminal body weights. Data are presented as mean ± SD. No significant between-group difference was observed. (n=6 each group)

Log2 ratio (DR/DNR)	Gene name	Gene description
4.556366	Yy2	Yy2 transcription factor
3.351245	Ugt8a	UDP galactosyltransferase 8A
2.658435	Zfp82	zinc finger protein 82

2.088528	Btnl7-ps	"butyrophilin-like 7, pseudogene
1.817854	Apold1	apolipoprotein L domain containing 1
1.653878	Olfr78	olfactory receptor 78
1.362057	Gins2	GINS complex subunit 2
1.333212	Lep	leptin
1.287274	Mal	myelin and lymphocyte protein, T cell differentiation protein
1.182015	Mpz	myelin protein zero
1.174729	Flrt1	fibronectin leucine rich transmembrane protein 1
1.125886	Prx	periaxin
1.125371	Mbp	myelin basic protein
1.117575	Bcl6b	B cell CLL/lymphoma 6, member B
1.056045	Ciart	circadian associated repressor of transcription
1.016935	Dach2	dachshund family transcription factor 2
1.016062	Lmod1	leiomodin 1
1.004962	Zc3h6	zinc finger CCCH type containing 6

Supplementary Table S2. The genes in the resistance exercise group (DR) had the greatest upregulation compared with muscles without exercise (DNR).

Log2 ratio (DR/DNR)	Gene name	Gene description
-12.7728	Ifitm6	interferon induced transmembrane protein 6
-11.1516	Ska3	spindle and kinetochore associated complex subunit 3
-11.0521	Spic	Spi-C transcription factor
-10.6495	Alb	albumin
-10.2307	Slc39a12	solute carrier family 39, member 12
-10.102	Adgre4	adhesion G protein-coupled receptor E4
-3.31437	Igkc	immunoglobulin kappa constant
-2.87123	Angptl4	angiopoietin-like 4
-2.12408	Pdk4	pyruvate dehydrogenase kinase, isoenzyme 4
-2.07238	Ces2c	carboxylesterase 2C

-1.66212	Rps15a-ps3	ribosomal protein S15A, pseudogene 3
-1.60822	Ucp3	uncoupling protein 3 (mitochondrial, proton carrier)
-1.56408	Mt2	metallothionein 2
-1.51542	Mki67	antigen identified by monoclonal antibody Ki 67
-1.31569	Foxo1	forkhead box O1
-1.30992	Mfap4	microfibrillar-associated protein 4
-1.22548	Ifi27l2a	"interferon, alpha-inducible protein 27 like 2A
-1.21837	CCL 6	chemokine (C-C motif) ligand 6
-1.20731	Actc1	"actin, alpha, cardiac muscle 1
-1.18931	Tnmd	tenomodulin

Supplementary Table S3. The genes in the resistance exercise group (DR) had the greatest degree of downgrading compared with muscles without exercise (DNR).

Log2 ratio (DR/DNR)	Gene name	Gene description
-15.6269	S100a9	S100 calcium binding protein A9
-14.095	S100a8	S100 calcium binding protein A8
-13.29	Camp	cathelicidin antimicrobial peptide
-11.6884	CCL 12	chemokine (C-C motif) ligand 12
-10.747	H2-Oa	"histocompatibility 2, O region alpha locus
-10.0705	Ibsp	integrin binding sialoprotein
-2.4393	Cd55b	"CD55 molecule, decay accelerating factor for complement B
-2.31587	Csf3r	colony stimulating factor 3 receptor (granulocyte)
-2.19366	CCR 1	chemokine (C-C motif) receptor 1
-2.10813	Ccnb2	cyclin B2
-1.99124	Krt18	keratin 18
-1.9805	Bmp8a	bone morphogenetic protein 8a
-1.93233	Lcn2	lipocalin 2
-1.81794	CCR 5	chemokine (C-C motif) receptor 5
-1.80052	Gapdh	glyceraldehyde-3-phosphate dehydrogenase

-1.7723	Cxcl13	chemokine (C-X-C motif) ligand 13
-1.73506	Aldoc	"aldolase C, fructose-bisphosphate
-1.58375	Dmp1	dentin matrix protein 1
-1.57815	CCL 21b	chemokine (C-C motif) ligand 21B (leucine)
-1.51854	Plk1	polo like kinase 1
-1.3943	Col2a1	collagen, type II, alpha 1
-1.37577	Col1a1	"collagen, type I, alpha 1
-1.31569	Foxo1	forkhead box O1
-1.25712	Col1a2	"collagen, type I, alpha 2
-1.25396	C1s2	"complement component 1, s subcomponent 2
-1.21837	CCL 6	chemokine (C-C motif) ligand 6
-1.11959	Cx3cl1	chemokine (C-X3-C motif) ligand 1
-1.10963	Pck1	"phosphoenolpyruvate carboxykinase 1, cytosolic
-1.03561	CCL 9	chemokine (C-C motif) ligand 9
-1.01012	Il17re	interleukin 17 receptor E
-1.00705	Selplg	"selectin, platelet (p-selectin) ligand
-1.00626	Itgam	integrin alpha M
1.111392	Plk3	polo like kinase 3
1.333212	Lep	leptin
1.377416	Fosb	FBJ osteosarcoma oncogene B
11.33773	Tnfrsf18	"tumor necrosis factor receptor superfamily, member 18
-11.0521	Spic	Spi-C transcription factor (Spi-1/PU.1 related)

Supplementary Table S4. The influence of muscle and inflammation-related genes in the resistance exercise group (DR) compared to the non-exercise (DNR) group.
